# Analysis of Daily Visual Habits in a Presbyopic Population

**DOI:** 10.1155/2023/6440954

**Published:** 2023-04-08

**Authors:** Filomena Ribeiro, Tiago B. Ferreira, Diana Silva, Ana Cláudia Matos, Sylvia Gaspar, David P. Piñero

**Affiliations:** ^1^Hospital da Luz Lisboa, Lisbon, Portugal; ^2^Lisbon University, Lisbon, Portugal; ^3^Visual Sciences Research Centre, Lisbon, Portugal; ^4^Department of Optics Pharmacology and Anatomy, University of Alicante, Alicante, Spain

## Abstract

**Purpose:**

To evaluate and report the visual habits and requirements of a sample of presbyopic patients using an advanced sensor.

**Methods:**

Transversal study collecting clinical data from 40 presbyopes candidates for presbyopia-correction intraocular lens (IOL) implantation with mean age of 61.0 years (43–80 years). A complete ophthalmological examination was performed in all patients including visual, refractive, an ocular biometric analysis. Furthermore, patients were instructed about the use of the Vivior Monitor system (Vivior AG, Zürich, Switzerland), which consists of a series of sensors attached to the rim of the patient's glasses that capture information about the visual behavior of the patient. This device was worn for a period of 36 hours or more. The data collected were transferred to a database and analyzed.

**Results:**

Mean percentages of time dedicated to distance, intermediate, and near vision were 27.25 ± 11.93% (5–65%), 30.23 ± 9.36% (12–50%), and 42.53 ± 14.96% (13–78%), respectively. Mean percentages of time performing activities under photopic, mesopic, and scotopic conditions were 37.08 ± 23.20% (5–87%), 33.65 ± 13.84% (6–67%), and 29.28 ± 17.03% (4–65%). The percentage of time with digital screens ranged from 2% to 48%. Age was significantly correlated with the percentage of time dedicated to distance vision (*r* = 0.317, *p*=0.047) and to activities performed under photopic conditions (*r* = −0.344, *p*=0.030).

**Conclusions:**

Distance and illumination conditions used to perform different daily life visual activities vary significantly among presbyopes, with a trend to the dedication of more time to intermediate and near visual activities performed under photopic and mesopic conditions. Data interpretation should be done with care until a proper validation of the device used.

## 1. Introduction

Presbyopia is a condition related to the aging of the eye consisting of a progressive worsening of the ability of the eye to focus clearly on intermediate and near objects [1]. Uncorrected presbyopia is a source of significant burden of visual impairment and for this reason its correction is mandatory [1], having a significant impact on the quality of life [2]. Cataract surgery with implantation of a presbyopia-correcting (PC) IOL is one opportunity for presbyopia correction [3–5]. This type of implants generates multiple foci or a continuous range of vision allowing patients to perform their daily activities at different distances [3–6]. The optical design and performance of multifocal IOLs are directly related to the range of functional vision that can provide [7, 8]. For this reason, the daily visual demands of patients should be investigated preoperatively and considered for the selection of the most adequate PC intraocular implant in each specific case. Gil et al. [9] concluded in a comparative study of four different multifocal IOLs that their characteristics evaluated in terms of optics, profile, and add power may contribute to help surgeons decide on the type of IOL most suitable for each patient, especially those with high visual demands at near and intermediate distances. Cillino and colleagues [10] concluded in another comparative study of three multifocal IOLs that intrinsic optical differences between them, such as optimization for computer or dim-light working or night driving, could be useful tools to customize the IOL in each single case.

Despite the relevance of the visual requirements of patients for the selection and implantation of PC IOLs, few studies have been conducted on this issue [11–17]. There are some studies that have evaluated the impact of the implantation of multifocal IOLs on reading distance [11–14], as well some studies analysing the distribution of the working distance and distance of use of electronic devices in presbyopic population [15–17]. Specifically, mean preferred reading distances between 32 cm and 41 cm has been reported in patients undergoing cataract surgery implanted with different modalities of multifocal IOL [11–14]. Furthermore, it has been demonstrated that there are some differences in visual demands and preferences among Asiatic and European individuals, being this another factor to consider [18, 19]. Recently, a new device capturing information about daily activities, working visual distances and illumination conditions has been developed (Vivior Monitor system, Vivior AG, Zürich, Switzerland), which can be especially useful for evaluating visual requirements and habits before and after cataract surgery with implantation of PC IOLs. It consists of a series of sensors measuring distance, ambient light and color, and blue light from digital screens. The aim of the current study was to evaluate and report for the first time using this technology the visual habits and requirements of a sample of presbyopic patients in the preoperative evaluation for surgery with implantation of a presbyopia correction IOL. This information may be especially useful for clinicians to understand visual patient's needs and meet patient's expectations providing spectacle independence for good postoperative functional vision, ultimately with an impact on quality of life.

## 2. Methods

### 2.1. Patients

This study was a transversal study resulting from the data of 40 patients collected prospectively on the preoperative screening of candidates for a surgery with the implantation of a presbyopia correction IOLs attending to our hospital. A total of 40 patients were recruited. The nature of the study was explained to all of them before their enrolment and signed the corresponding consent form prior to the initiation of measurements. The study adhered to the tenets of the Declaration of Helsinki and was approved by the ethics committee of the Hospital da Luz.

Inclusion criteria for the study were healthy patients with presbyopia (need for near addition of 0.75 D or more), clear crystalline lens or incipient cataract (corrected distance visual acuity of 20/60 or better), candidates for implantation of PC IOLs, no limitations to perform daily activities with their optical correction, and age between 42 and 80 years. Exclusion criteria included any active ocular disease, previous ocular surgery including laser refractive surgery, limited vision (corrected distance visual acuity of 20/60 or below), neurologic problems, strabismus, amblyopia, and no acceptance to wear the device for monitoring visual habits and illuminance conditions for at least 36 hours.

### 2.2. Clinical Protocol

A complete ophthalmological examination was performed in all patients that included measurement of uncorrected (UDVA) and corrected distance visual acuity (CDVA), manifest refraction, optical biometry and keratometry (Lenstar LS900, Haag-Streit AG, Koeniz, Switzerland), slit lamp examination, Goldman applanation tonometry, and fundus evaluation. In addition, age, height, body mass index, and profession were recorded as an additional variable to consider afterwards in the analysis. Furthermore, patients were instructed about the use of the Vivior Monitor to capture the information about their visual behavior.

The Vivior Monitor is a sensor about the size of a USB stick that was attached to the rim of the patient's glasses. This sensor allows measuring and monitoring visual distances and ambient light. It is important to remark that this device does not contain a camera, and therefore, its use does not infringe the privacy of patient and people around him/her. In the current study, this device was worn for a period of at least 36 hours. During the wearing period, the sensor collected data on viewing distance and light during everyday activities. All these data were analyzed by a cloud-based data processing system using artificial intelligence algorithms that proprietary of Vivior AG. The results obtained after this data processing were transferred to an Excel database, including the time the patient spent looking at far (>1 m), intermediate (0.5 to 1 m), and near distances (<0.5 m) under photopic, mesopic, and scotopic lighting conditions.

### 2.3. Statistical Analysis

Statistical analyses were performed with a commercially available software package (SPSS for Mac, Version 20.0; IBM Corporation, Armonk, NY, USA). Kolmogorov–Smirnov test was used to confirm the normality of the data distributions analysed. Measured variables (including percentages) were characterized with the following parameters: average, standard deviation, median, and range. The correlation of clinical variables and parameters measured with the sensor (including percentages) was analysed with the Pearson coefficient. The unpaired Student's *t* test was used for the comparison between independent groups (male-female). For all statistical tests, a *p* value below 0.05 was considered as statistically significant.

## 3. Results

A total of 40 presbyopic patients with mean age of 61.0 years (SD: 7.2, median: 61.0, range: 43 to 80 years) were evaluated. The sample was comprised of 17 males (42.5%) and 23 females (57.5%). Mean height and body mass index of patients evaluated were 163.77 cm (SD: 9.01, median: 164.00, range: 147.00 to 186.00 cm) and 24.95 kg/m^2^ (SD: 4.22, median: 24.00, range: 17.78 to 35.82 kg/m^2^), respectively.  Table 1 summarizes the main clinical data obtained in the sample evaluated.

### 3.1. Visual Behaviour Characterized with the Sensor

**Figure 1:**
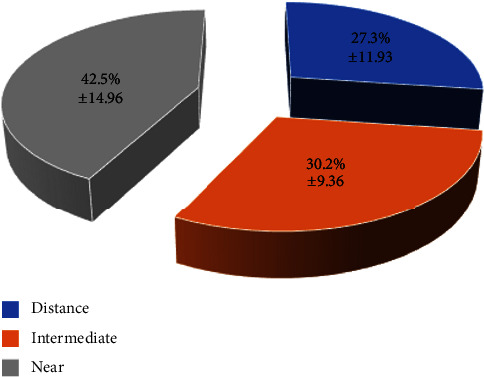
Mean percentage of the time dedicated to distance, intermediate, and near visual activities measured in the evaluated sample.

**Figure 2:**
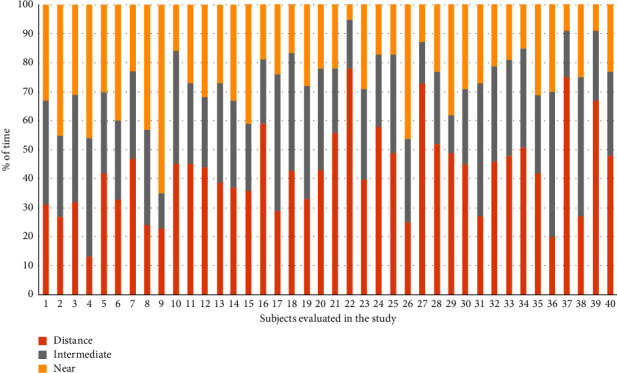
Distribution per patient of the time dedicated to distance, intermediate, and near visual activities measured in the evaluated sample.

Figure 1 shows the mean times dedicated to distance, intermediate, and near visual activities in the evaluated sample. The percentage of time dedicated to distance vision ranged from 5 to 65% (mean: 27.25; SD: 11.93; median: 27.00), whereas the percentage of time dedicated to near visual activities ranged from 13 to 78% (mean: 42.53; SD: 14.96; median: 43.00). Concerning intermediate vision, this percentage ranged from 12 to 50% (mean: 30.23; SD: 9.36; median: 29.50). More than 30% of time was dedicated to distance, intermediate, and near in 32.5%, 50%, and 77.5% of patients, respectively (Figure 2).

**Figure 3:**
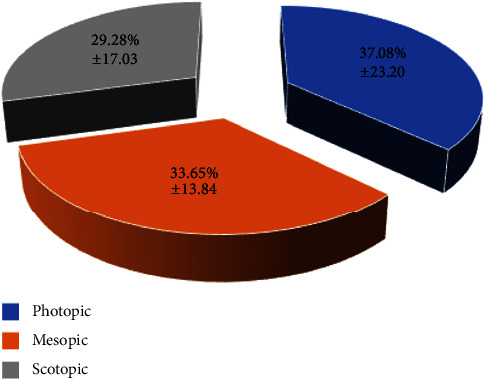
Mean percentage of the time dedicated to activities performed under photopic, mesopic, and scotopic conditions measured in the evaluated sample.

**Figure 4:**
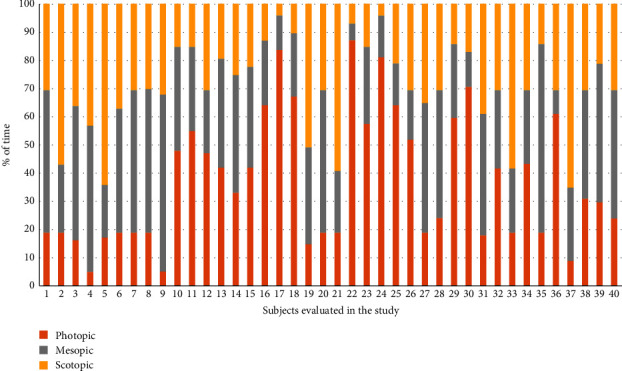
Distribution per patient of the time dedicated to activities performed under photopic, mesopic, and scotopic conditions measured in the evaluated sample.

Figure 3 shows the mean times dedicated to activities under photopic, mesopic, and scotopic conditions in the evaluated sample. The percentage of time performing activities under photopic conditions ranged from 5 to 87% (mean: 37.08; SD: 23.20; median: 30.50), whereas the percentage of time dedicated to activities performed under scotopic conditions ranged from 4 to 65% (mean: 29.28; SD: 17.03; median: 29.50). Concerning activities under mesopic conditions, this percentage of time dedicated to them ranged from 6 to 67% (mean: 33.65; SD: 13.84; median: 33.00). More than 30% of time was dedicated to activities performed under photopic, mesopic, and scotopic conditions in 50%, 57.5%, and 42.5% of patients, respectively (Figure 4).

Mean percentage of time with digital screens in the evaluated sample was 18.98% (SD: 10.48; median: 19.00%), with values ranging from 2% to 48%.

### 3.2. Relationship between Age, Anthropometric Data, Professional Activity, Visual Habits, and Illumination Conditions

Age was significantly correlated with the percentage of time dedicated to distance vision (*r* = 0.317, *p*=0.047) and with the percentage of time dedicated to activities performed under photopic conditions (*r* = −0.344, *p*=0.030), although the correlations were weak. In addition, a weak but statistically significant correlation was found between the percentage of time dedicated to distance vision and that dedicated to activities performed under photopic conditions (*r* = −0.316, *p*=0.047). The percentage of time dedicated to activities performed under mesopic conditions was also significantly correlated with the percentages of time associated to distance (*r* = 0.384, *p*=0.015) and near vision (*r* = −0.339, *p*=0.032).

**Figure 5:**
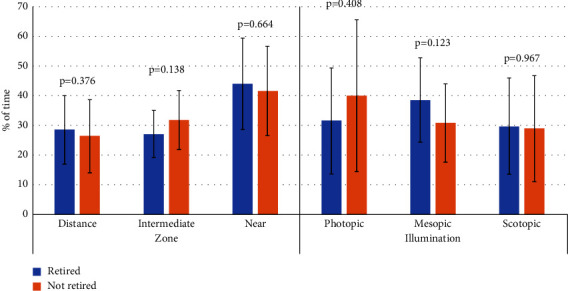
Mean percentages of time dedicated to distance, intermediate, and near vision and to activities performed under photopic, mesopic, and scotopic conditions in retired and not retired patients.

There was no correlation of IMC with the percentage of time dedicated to distance (*r* = 0.023, *p*=0.890), intermediate (*r* = −0.233, *p*=0.153) and near vision (*r* = 0.041, *p*=0.806). This parameter did not correlate either with the percentage of time dedicated to activities performed under photopic (*r* = −0.105, *p*=0.524), mesopic (*r* = −0.009, *p*=0.956) and scotopic conditions (*r* = 0.202, *p*=0.216). Concerning height, it was not significantly correlated with the percentages of time dedicated to different distances (distance: *r* = 0.052, *p*=0.755; intermediate: *r* = 0.231, *p*=0.158; near: *r* = −0.207, *p*=0.207) and illumination conditions (photopic: *r* = 0.093, *p*=0.572; mesopic: *r* = 0.218, *p*=0.183; near: *r* = −0.157, *p*=0.340). Finally, no significant differences in the distribution of time dedicated to distance, intermediate, and near vision as well as in the percentages of time performing activities under photopic, mesopic, and scotopic conditions were found between subjects retired and not retired (Figure 5) (*p* ≥ 0.123).

## 4. Discussion

Patient selection is a crucial issue when recommending the implantation of a specific model of PC IOL [6]. The optical performance of each specific model of PC IOL according to its design should be known and considered by the practitioner before any type of recommendation as the range of tolerable visual quality and foci may vary significantly between IOL models [20]. The daily patient's visual demands and requirements should be considered in the attempt of finding the PC IOL model providing the range of focal distances matching better with the specific range of distances commonly used by patients for performing their daily life activities. Several studies have been conducted to characterize optically and clinically the outcomes obtained with a great variety of PC IOLs [3, 4, 6–12, 20–29], but the scientific literature on the distribution of common patient's visual demands and range of focus required is still scarce [13–15, 30]. Furthermore, there are few clinical tools to evaluate these aspects, being most of them time consuming. For this reason, an investigation on this area is necessary, especially considering that there is new technology allowing an objective characterization of visual habits. This study was aimed at evaluating and reporting using the Vivior Monitor technology the visual habits and requirements of a sample of presbyopic patients. An additional aim was to investigate the relationship between these visual habits and different clinical variables in order to define new clinical guidelines for specific subgroups of patients.

In the sample evaluated, most part of time of the presbyopic patients monitored was dedicated to near (42.53%) and intermediate visual activities (30.23%), which confirms the relevance of the range of distances between 1 m and approximately 30–40 cm for the daily life activities of presbyopes. This result is consistent with those from a previous transversal study showing a mean working distance of 82.5 cm and a mean mobile phone usage distance of 31.9 cm in a sample of 454 participants with a mean age of 41.5 years (range, 22–64 years) [14]. Likewise, Boccardo [15] reported recently in another cross-sectional observational study a mean smartphone viewing distance of 35.0 ± 6.4 cm in a group of nonpresbyopic patients and a mean value of 39.0 ± 6.1 cm in a group of presbyopes. Therefore, the measurement of distance and near visual acuity at 40 cm is not sufficient to evaluate if the visual performance achieved by a patient implanted with a PC IOL is optimum or not. As recently suggested by a group of experts, the concept of functional vision range should be considered and compared with the range of patient's visual demands to determine the level of matching between both, and consequently, the adequacy of the implant [31]. Surgeons are commonly used to measure visual outcomes for far and near distances, but in the current series the use of intermediate vision has been shown to be crucial and must be considered. This may be in relation with the new visual habits associated to the digital era, with more time spent working with the computer and tablets [32].

Although the general trend of the sample evaluated was the dedication of more time to intermediate and near visual activities, a great variability was observed in the distribution of time dedicated to distance, intermediate, and near among individuals. This means that this relationship cannot be predicted with accuracy. Indeed, the percentage of time dedicated to distance vision ranged from 5 to 65%, whereas the ranges for the percentages of time dedicated to intermediate and near visual activities were 12 to 50% and 13 to 78%, respectively. Furthermore, no significant correlations were found between these percentages and different clinical variables evaluated, not allowing to obtain subgroups of patients with specific profiles to be considered in the preoperative screening for PC IOL implantation. Therefore, the characterization of the visual habits should be performed in the preoperative exam for cataract surgery with implantation of PC IOL to detect the real patient's visual demands and to find the implant providing the foci covering such demands.

Besides the time dedicated to distance, intermediate, and near visual acuities, a characterization of the illumination conditions was also provided by the Vivior Monitor in the current study, obtaining a surprising trend to more time dedicated to visual activities under photopic (37.08%) and mesopic (33.65%) conditions than under scotopic (29.28%) conditions. However, the level of variability in this distribution of illumination conditions also varied significantly among individuals, with a range of variation of the time dedicated to activities under photopic, mesopic, and scotopic conditions of 5 to 87%, 6 to 67%, and 4 to 65%, respectively. This factor is especially critical considering that eyes implanted with multifocal IOLs had a photopic pupil size of 3.5 mm or less and a mesopic pupil size of 5 mm or less [33], and this change can be very relevant clinically when they are implanted with IOL models showing some type of pupillary dependence or leading to a decrease in contrast sensitivity especially in mesopic conditions [34, 35]. Therefore, a characterization of the illumination conditions, and not only of distance, should be considered as an additional critical factor when selecting the specific type of PC IOL to implant in each case.

Finally, weak but statistically significant correlations of age with the percentage of time dedicated to distance vision (*r* = 0.317) and with the percentage of time dedicated to activities performed under photopic conditions (*r* = −0.344) were found. These correlations indicated a trend of patients to dedicate more time to distance vision and less time to perform activities under photopic conditions with increasing age. This may be related to the changes in lifestyle with age, with potentially more time dedicated to activities at home using artificial lights and less time dedicated to activities with increasing difficulties, but this should be investigated in future studies. Besides this, weak but statistically significant correlations of the percentage of time dedicated to activities performed under mesopic conditions with the percentages of time associated to distance (*r* = 0.384) and near vision (*r* = −0.339) were also found. This means that there was a trend to an increase of the use of distance vision under mesopic conditions, with less near visual activities performed under these illumination conditions. This seems coherent considering that one of the most important activities performed at near is reading, for which good illumination is needed. Furthermore, the mean percentage of time dedicated to activities with digital screens was almost 19% in the sample evaluated, although this percentage ranged from 2% to 48%. This confirms that a great proportion of subjects dedicated a relevant part of their time to the use of digital screens, which is consistent with changes in visual habits associated to the digital era, with significantly more time using computers and tablets [32, 36]. Future studies with larger samples should confirm all these trends that might be considered in future IOL design developments as well as in clinical practice.

This study has some limitations that must be acknowledged. The most important limitation of the current study and consequently its main weakness is the lack of proper validation of the Vivior device. To this date, there are no published reports about the reliability of this instrument used to characterize the daily visual habits and consequently some level of bias could be present in the data obtained in the study and presented here. This means that data interpretation and extrapolation should be done with care. The data provided should be considered as preliminary trends to confirm in the future. However, to this date, the Vivior system is the only instrument of clinical use, which is commercially available for measuring and characterizing daily visual habits. Future studies should be conducted to confirm the reliability and usefulness of this new technology. Another important limitation of the study is the selection criteria and the limited sample size, which supposes that this information cannot be systematically extrapolated to the general population. This investigation should be considered as a first trial showing the trends in daily visual habits in a specific subsample of the presbyopic population. In any case, as previously commented, a huge variation in visual behavior and also regarding the use of light was found that makes the interpretation and generalization extremely difficult. Future studies are needed to corroborate those findings and this high variation in daily visual habits among presbyopes.

In conclusion, distance and illumination conditions used to perform different daily life visual activities vary significantly among presbyopes, with no predictable relationship between them, although this should be confirmed in detail after a proper validation of the device used. In any case, there is a trend in presbyopes to the dedication of more time to intermediate and near visual activities performed under photopic and mesopic conditions. This remarks the relevance of the intermediate vision in daily life activities. In addition, it is important to consider that a relevant percentage of time is dedicated to mesopic activities. Considering this, it would be highly recommendable a preoperative assessment of visual distance and illumination habits in patients that are going to undergo transparent crystalline lens surgery for the compensation of presbyopia. According to this and if the reliability of the measurements provided by the system used is confirmed, this procedure of characterizing daily visual habits would be crucial for an appropriate selection of the most adequate PC IOL to implant. A personalization of the implant could be performed according to an analysis not only including standard clinical variables but also visual habits. Distance and near (40 cm) visual acuity should no longer be the only variables to measure the performance of a presbyopia correcting IOL and to understand the patient satisfaction after implantation. The measurement of functional vision should also address the performance of the IOL for intermediate and closer than 40 cm distance, as well as the performance of the IOL under different types of lighting conditions.

## Figures and Tables

**Table 1:** Summary of the main clinical data obtained in the sample of patients evaluated.

Mean (SD)	Right eye	Left eye
Median (range)		
Sphere (D)	−0.28 (3.08)	−0.06 (3.06)
	0.63 (−10.75–3.25)	1.00 (−9.50–4.50)

Cylinder (D)	−0.79 (0.94)	−0.53 (0.87)
	−0.50 (−3.00–0.00)	−0.38 (−3.25–0.00)

SE (D)	−0.68 (3.28)	−0.33 (3.31)
	0.56 (−11.75–3.00)	0.94 (−10.38–4.25)

K1 (D)	43.18 (2.13)	43.29 (2.02)
	43.13 (39.55–49.67)	42.94 (40.12–49.46)

K2 (D)	44.07 (2.04)	44.04 (1.98)
	43.66 (40.91–50.00)	43.64 (41.06–49.80)

CA (D)	0.97 (0.89)	0.83 (0.68)
	0.60 (0.04–3.39)	0.66 (0.05–2.99)

WTW (mm)	12.00 (0.40)	12.01 (0.36)
	12.03 (11.06–12.70)	12.03 (11.20–12.66)

AL (mm)	23.93 (1.47)	23.77 (1.44)
	23.80 (21.53–27.51)	23.70 (21.41–27.09)

CCT (*μ*m)	543.72 (21.82)	543.69 (22.00)
	547.50 (498.00–579.00)	548.00 (499.00–584.00)

ACD (mm)	2.84 (0.41)	2.80 (0.40)
	2.86 (1.98–3.88)	2.76 (1.98–3.73)

LT (mm)	4.31 (0.35)	4.33 (0.33)
	4.36 (3.24–4.96)	4.33 (3.47–5.02)

% Time dedicated to distance vision	27.25 (11.93)	
	27.00 (5 to 65)	

% Time dedicated to intermediate vision	30.23 (9.36)	
	29.50 (12– 50)	

% Time dedicated to near vision	42.53 (14.96)	
	43.00 (13–78)	

% Time performing activities under photopic conditions	37.08 (23.20)	
	30.50 (5– 87)	

% Time performing activities under mesopic conditions	33.65 (13.84)	
	33.00 (6–67)	

% Time performing activities under scotopic conditions	29.28 (17.03)	
	29.50 (4–65)	

% Time with digital screens	18.98 (10.48)	
	19.00 (2–48)	

Abbreviations: SD, standard deviation; SE, spherical equivalent; K1, flattest keratometric reading; K2, steepest keratometric reading; CA, corneal astigmatism; WTW, white-to-white corneal diameter; AL, axial length; CCT, central corneal thickness; ACD, anterior chamber depth; LT, lens thickness.

## Data Availability

The data used to support the findings of this study are available from the corresponding authors on reasonable request.

## References

[1] Fricke T. R., Tahhan N., Resnikoff S. (2018). Global prevalence of presbyopia and vision impairment from uncorrected presbyopia: systematic review, meta-analysis, and modelling. *Ophthalmology*.

[2] Sivardeen A., McAlinden C., Wolffsohn J. S. (2020). Presbyopic correction use and its impact on quality of vision symptoms. *Journal of Optometry*.

[3] Khandelwal S. S., Jun J. J., Mak S., Booth M. S., Shekelle P. G. (2019). Effectiveness of multifocal and monofocal intraocular lenses for cataract surgery and lens replacement: a systematic review and meta-analysis. *Graefes Archive for Clinical and Experimental Ophthalmology*.

[4] Ribeiro F. J., Ferreira T. B., Silva D., Matos A. C., Gaspar S. (2021). Visual outcomes and patient satisfaction after implantation of a presbyopia-correcting intraocular lens that combines extended depth-of-focus and multifocal profiles. *Journal of Cataract & Refractive Surgery*.

[5] Ferreira T. B., Ribeiro F. J., Silva D., Matos A. C., Gaspar S., Almeida S. (2022). Comparison of refractive and visual outcomes of 3 presbyopia-correcting intraocular lenses. *Journal of Cataract & Refractive Surgery*.

[6] Akella S. S., Juthani V. V. (2018). Extended depth of focus intraocular lenses for presbyopia. *Current Opinion in Ophthalmology*.

[7] Fernández J., Rodríguez-Vallejo M., Martínez J., Burguera N., Piñero D. P. (2019). Prediction of visual acuity and contrast sensitivity from optical simulations with multifocal intraocular lenses. *Journal of Refractive Surgery*.

[8] Plaza-Puche A. B., Alió J. L., MacRae S., Zheleznyak L., Sala E., Yoon G. (2015). Correlating optical bench performance with clinical defocus curves in varifocal and trifocal intraocular lenses. *Journal of Refractive Surgery*.

[9] Gil M. A., Varon C., Rosello N., Cardona G., Buil J. A. (2012). Visual acuity, contrast sensitivity, subjective quality of vision, and quality of life with 4 different multifocal IOLs. *European Journal of Ophthalmology*.

[10] Cillino G., Casuccio A., Pasti M., Bono V., Mencucci R., Cillino S. (2014). Working-age cataract patients: visual results, reading performance, and quality of life with three diffractive multifocal intraocular lenses. *Ophthalmology*.

[11] Linz K., Attia M. S. A., Khoramnia R., Tandogan T., Kretz F. T., Auffarth G. U. (2016). Clinical evaluation of reading performance using the Salzburg Reading Desk with a refractive rotational asymmetric multifocal intraocular lens. *Journal of Refractive Surgery*.

[12] Rasp M., Bachernegg A., Seyeddain O. (2012). Bilateral reading performance of 4 multifocal intraocular lens models and a monofocal intraocular lens under bright lighting conditions. *Journal of Cataract & Refractive Surgery*.

[13] Alió J. L., Grabner G., Plaza-Puche A. B. (2011). Postoperative bilateral reading performance with 4 intraocular lens models: six-month results. *Journal of Cataract & Refractive Surgery*.

[14] Alió J. L., Plaza-Puche A. B., Piñero D. P. (2011). Optical analysis, reading performance, and quality-of-life evaluation after implantation of a diffractive multifocal intraocular lens. *Journal of Cataract & Refractive Surgery*.

[15] Soler F., Sánchez-García A., Molina-Martín A., de Fez D., Díaz V., Piñero D. P. (2021). Analysis of the characteristics of electronic equipment usage distance for common users. *Guoji Yanke Zazhi*.

[16] Soler F., Sánchez-García A., Molina-Martín A., de Fez D., Díaz V., Piñero D. P. (2021). Differences in visual working and mobile phone usage distance according to the job profile. *Current Eye Research*.

[17] Boccardo L. (2021). Viewing distance of smartphones in presbyopic and non-presbyopic age. *Journal of Optometry*.

[18] Zhang J., Liu J., Jasti S., Suryakumar R., Bullimore M. A. (2020). Visual demand and acuity reserve of Chinese versus English newspapers. *Optometry and Vision Science*.

[19] Negishi K., Hayashi K., Kamiya K. (2019). Nationwide prospective cohort study on cataract surgery with multifocal intraocular lens implantation in Japan. *American Journal of Ophthalmology*.

[20] Alió J., Salerno L., Tiveron M. (2017). Multifocal intraocular lenses: types, outcomes, complications and how to solve them. *Taiwan Journal of Ophthalmology*.

[21] Reinhard T., Maier P., Böhringer D. (2021). Comparison of two extended depth of focus intraocular lenses with a monofocal lens: a multi-centre randomised trial. *Graefes Archive for Clinical and Experimental Ophthalmology*.

[22] Ribeiro F., Ferreira T. B. (2020). Comparison of clinical outcomes of 3 trifocal IOLs. *Journal of Cataract & Refractive Surgery*.

[23] Kohnen T., Marchini G., Alfonso J. F. (2020). Innovative trifocal (quadrifocal) presbyopia-correcting IOLs: 1-year outcomes from an international multicenter study. *Journal of Cataract & Refractive Surgery*.

[24] Royo M., Jiménez Á., Piñero D. P. (2020). Clinical outcomes of cataract surgery with implantation of a continuous transitional focus intraocular lens. *Journal of Cataract & Refractive Surgery*.

[25] Lapid-Gortzak R., Bhatt U., Sanchez J. G. (2020). Multicenter visual outcomes comparison of 2 trifocal presbyopia-correcting IOLs: 6-month postoperative results. *Journal of Cataract & Refractive Surgery*.

[26] Auffarth G. U., Moraru O., Munteanu M. (2020). European, multicenter, prospective, non-comparative clinical evaluation of an extended depth of focus intraocular lens. *Journal of Refractive Surgery*.

[27] Webers V. S. C., Bauer N. J. C., Saelens I. E. Y. (2020). Comparison of the intermediate distance of a trifocal IOL with an extended depth-of-focus IOL: results of a prospective randomized trial. *Journal of Cataract & Refractive Surgery*.

[28] Pedrotti E., Chierego C., Talli P. M. (2020). Extended depth of focus versus monofocal IOLs: objective and subjective visual outcomes. *Journal of Refractive Surgery*.

[29] Böhm M., Petermann K., Hemkeppler E., Kohnen T. (2019). Defocus curves of 4 presbyopia-correcting IOL designs: diffractive panfocal, diffractive trifocal, segmental refractive, and extended-depth-of-focus. *Journal of Cataract & Refractive Surgery*.

[30] Bartha M. C., Allie P., Kokot D., Roe C. P. (2015). Field observations of display placement requirements and character size for presbyopic and prepresbyopic computer users. *Work*.

[31] Ribeiro F., Cochener B., Kohnen T. (2020). Definition and clinical relevance of the concept of functional vision in cataract surgery. ESCRS position statement on intermediate vision ESCRS functional vision working group. *Journal of Cataract & Refractive Surgery*.

[32] Ofcom (2018). Adults’ Media Use and Attitudes Report. https://www.ofcom.org.uk/__data/assets/pdf_file/0011/113222/Adults-Media-Use-and-Attitudes-Report-2018.pdf.

[33] Fernández J., Rodríguez-Vallejo M., Martínez J., Burguera N., Piñero D. P. (2020). Pupil diameter in patients with multifocal intraocular lenses. *Journal of Refractive Surgery*.

[34] Messias A., Ferreira M., Mendonça G. C., Queiroz W., Coelho R. P., Gekeler K. (2021). Influence of pupillary dynamics on the defocus curve of eyes implanted with diffractive multifocal lenses: a randomized study. *Arquivos Brasileiros de Oftalmologia*.

[35] García-Domene M. C., Felipe A., Peris-Martínez C., Navea A., Artigas J. M., Pons A. M. (2015). Image quality comparison of two multifocal IOLs: influence of the pupil. *Journal of Refractive Surgery*.

[36] De-Sola J., Rubio G., Talledo H., Pistoni L., Van Riesen H., Rodríguez de Fonseca F. (2019). Cell phone use habits among the Spanish population: contribution of applications to problematic use. *Frontiers in Psychiatry*.

[37] Ribeiro F., Ferreira T. B., Silva D., Cláudia M. A., Sylvia G., Piñero D. P. (2022). Analysis of daily visual habits in a presbyopic population. *Research Square*.

